# Risk of stomach cancer incidence in a cohort of Mayak PA workers occupationally exposed to ionizing radiation

**DOI:** 10.1371/journal.pone.0231531

**Published:** 2020-04-15

**Authors:** Galina V. Zhuntova, Tamara V. Azizova, Evgeniya S. Grigoryeva

**Affiliations:** Clinical Department, Southern Urals Biophysics Institute (SUBI), Ozyorsk, Chelyabinsk region, Russia; Kagoshima University Graduate School of Medical and Dental Sciences, JAPAN

## Abstract

Stomach cancer is a widespread health condition associated with environmental and genetic factors. Contribution of ionizing radiation to stomach cancer etiology is not sufficiently studied. This study was aimed to assess an association of the stomach cancer incidence risk with doses from occupational radiation exposure in a cohort of workers hired at main Mayak production association facilities in 1948–1982 taking into account non-radiation factors including digestive disorders. The study cohort comprised 22,377 individuals and by 31.12.2013 343 stomach cancer diagnoses had been reported among the cohort members. Occupational stomach absorbed doses were provided by the Mayak Worker Dosimetry System– 2008 (*MWDS–2008*) for external gamma ray exposure and by the Mayak Worker Dosimetry System– 2013 (*MWDS–2013*) for internal exposure to plutonium. Excess relative risks (ERR) per Gy for stomach cancer were estimated using the Poisson’s regression. Analyses were run using the AMFIT module of the EPICURE software. The stomach cancer incidence risk in the study cohort was found to be significantly associated with the stomach absorbed dose of gamma rays: ERR/Gy = 0.19 (95% CI: 0.01, 0.44) with a 0 year lag, and ERR/Gy = 0.20 (95% CI: 0.01, 0.45) with a 5 year lag. To estimate the baseline risk, sex, attained age, smoking status and alcohol consumption, chronic diseases (peptic ulcer, gastritis and duodenitis) were taken into account. No modifications of the radiogenic risk by non-radiation factors were found in the study worker cohort. No association of the stomach cancer incidence risk with internal exposure to incorporated plutonium was observed.

## Introduction

Stomach cancer (StoCa) remains one of the most frequent malignant neoplasms despite decreasing incidence and mortality rates observed during recent decades in many countries [1]. StoCa is a polyetiological disease that develops as a result of a complex interaction of environmental factors and the level of the effect of this interaction considerably depends on genetically determined features of an organism. *Helicobacter pylori* (*H*. *pylori*) infection, unhealthy diet, smoking, alcohol consumption have been recognized to be main risk factors for StoCa [2–6].

Epidemiological studies of the Japanese atomic-bomb survivors [7, 8] and patients who had undergone radiation therapy [9–13] demonstrated increased risks of StoCa incidence and mortality related to ionizing radiation exposure. Large studies of nuclear workers [14, 15] or healthcare staff [16] did not demonstrate convincing evidence of the StoCa association with occupational radiation (mostly, low dose) exposure.

The cohort of the first Russian nuclear production facility, the Mayak Production association, demonstrated increased radiogenic risks of malignant neoplasms, however data on StoCa are inconsistent: incidence and mortality analyses (follow-up periods of 1948–2004 and 1948–2008, respectively) did reveal increasing excess relative risks for StoCa, but the association with dose from external radiation did not reach statistical significance [17, 18]. A case-control study [19] demonstrated that in addition to external gamma-ray exposure, smoking and alcohol consumption, chronic digestive disorders have a considerable effect on the incidence rate of StoCa among Mayak PA workers but potential contribution of these factors was not taken into account in previous studies of cancer outcomes in the cohort [17, 18].

The follow-up of Mayak PA workers now has been extended, improved and updated estimates of alpha radiation doses from internal exposure have become available [20], detailed information on morbidity of the workers have been accumulated–all these achievements enable updating the results of previous studies of occupational radiation exposure and the StoCa risk.

The present study was aimed to assess the StoCa incidence risk association with doses from occupational radiation exposure for Mayak PA workers first hired at main production facilities in 1948–1982 taking into account non-radiation factors and chronic digestive disorders among them.

## Materials and methods

The present record-based epidemiological study did not require any contact with cohort members. The study was reviewed and approved by the Institutional Review Board (IRB) of the Southern Urals Biophysics Institute. SUBI IRB confirmed that no signed consents were needed from members of the study cohort. The study was performed in accordance with the Declaration of Helsinki.

### Study cohort

The present retrospective cohort study considered workers of the Mayak PA, the first Russian industrial facility that started operations in late 40s of the XX century to produce weapon-grade plutonium and fission products. The cohort comprised 22,377 individuals (with 25% of females) who were hired at main Mayak PA facilities (reactor, radiochemical and plutonium-production plants) in 1948–1982. As of 31.12.2013 vital status was ascertained for 21,258 (95%) of the cohort members—54% of them deceased and 46% were alive. Mean age (± standard deviation, SD) of death in males was 61.5±13.6 years, and in females it was 70.5±12.4 years while the mean age of alive workers was 68.5±10.4 years (in males) and 76.6±9.8 years (in females). 43 workers who had been acutely exposed to high doses of gamma rays or gamma-neutron radiation during radiation emergencies at the Mayak PA were excluded from the dataset for the study [21]. Additionally, 694 workers with missing medical records were also excluded. So, the present study included 20,521 workers first employed at the main Mayak PA facilities in 1948–1982 (hereinafter, ‘the study cohort’).

The follow-up of the study cohort members covered a period that started from a date of hire at one of the main facilities and ended on 31.12.2013 (a date of death / date of ‘the last registered medical information’ for those individuals who were lost to follow-up and for migrants either before 31.12.2013 or before stomach cancer was diagnosed). Data used in the present analysis were restricted to a period during which the workers were residing in Ozyorsk, the city located close to the Mayak PA, because information on diseases, results of annual health check-ups and non-radiation factors were unavailable for migrants after they had left the city.

Medical surveillance of workers residing in Ozyorsk was carried out in special health care facilities of the city. To be hired at the Mayak PA individuals mandatorily took a pre-employment medical examination aimed to assess an initial health status of a future worker. Later during employment, medical outpatient health checks were regularly carried out: before 1960 they were performed once in several months (due to high levels of radiation exposure of personnel), after 1960 (when the labor conditions considerably improved) they were performed once a year. Additionally, once every 5 years advanced medical health checks were performed at a specialized clinic. The program of medical follow-up developed and implemented specifically for Mayak PA workers as well as the program for collection and storage of medical data that enabled accumulation of high-quality information about workers health were described earlier [22, 23].

Data on smoking and alcohol consumption were collected and updated through interviewing the workers during regular medical heath checks. Workers’ responses to the questions were documented in medical records. For this study the category ‘heavy drinkers’ referred to those workers who had been treated from a drinking problem by an addiction medical specialist. Other categories for alcohol consumption ('non-drinkers', 'moderate drinkers') included individuals who provided a corresponding self-assessement for their habit of drinking alcohol.

By 31.12.2013 data on smoking status and alcohol consumption information over the whole follow-up has been available for 94% and 85% of the study cohort workers, respectively. Complete quantitative parameters of the smoking habit were available for a lesser number of workers (71%).

StoCa incidence data were taken from medical records of workers. The study considered all cases of StoCa that met the following criteria: StoCa was morphologically verified, or a tumor in a stomach was found during surgery, endoscopy or radiography (however, histological examination results are missing/no histological examination was performed) and a clinical pattern typical for the disease was observed with sequential signs of tumor progression.

The Russian Federation is among the countries with high prevalence of *H*. *pylori infection* [24]. Once this is a retrospective study covering a period over 60 years, it was impossible to provide a valid assessment of *H*. *pylori* infection contribution to StoCa occurrence in members of the study cohort. Diagnostic tests for *H*. *pylori* infection have been implemented in clinical practice only in recent decades and were carried out only for a limited number of workers as a diagnostics procedure. Meanwhile, chronic gastric diseases (stomach and duodenal ulcer, gastritis and duodenitis–ICD-10 codes K25–K26, K29) were taken into account for the analyses, and these diseases could be associated with *H*. *pylori* [25]. Information whether workers of the study cohort had these diseases or not was based on medical records.

Chronic gastric diseases were diagnosed with radiologic/endoscopic examinations, acid secretion tests, histological evaluation of gastric mucosa biopsy specimens and tests for *H*. *pylori* infection. Diagnostic tools for stomach diseases were changing during the considered period (1948–2013) and endoscopy was introduced only in the end of 1970s. It should be noted that radiology/endoscopy of stomach cancer was not mandatory for all cohort members and these procedures were carried out for diagnostic purposes in case of medical necessity after a worker had consulted a doctor.

The analysis of the radiogenic risk of StoCa took into account chronic gastric diseases as confounders if the diseases were diagnosed two years before the study exit date and earlier (for individuals diagnosed with StoCa not later than two years before a tumor was detected). This approach prevented from inclusion of undetected stomach tumors at early stages in the analysis as chronic stomach non-cancer disorders, both having similar symptoms at early stages.

### Dosimetry

About 55% of the study cohort members were hired at one of the main Mayak PA facilities in 1948–1958 when labor conditions at the enterprise were the worst what resulted in accumulation of high radiation doses by the personnel [26]. Almost 79% of the workers started their occupational history at the age before 30 years, and 59% of the cohort members were employed at the Mayak PA for more than 10 years.

Reactor workers (24% of the study cohort members) were exposed only to external gamma rays while radiochemical (41%) and plutonium production (35%) plant employees could have been exposed to alpha-active aerosols containing plutonium-239. Detailed description of the study cohort was published earlier [23].

For this study estimates of occupational radiation doses accumulated by workers by the end of the follow-up period were provided by the following dosimetry systems: cumulative stomach wall absorbed dose from external gamma rays (hereinafter, stomach gamma-doses) of the Mayak Worker Dosimetry System– 2008 (MWDS-2008) and cumulative stomach wall absorbed doses from internally deposited plutonium (hereinafter, stomach alpha-doses) of the Mayak Worker Dosimetry System– 2013 (MWDS-2013) [20, 26]. In the study cohort the mean stomach gamma-doses were 0.48 Gy (median 0.19 Gy, min–max 0.0–7.35 Gy) in males and 0.40 Gy (median 0.13 Gy, min-max 0.0–5.63 Gy) in females and the mean stomach alpha-doses were 0.0011 Gy (median 0.0002 Gy, min–max 0.0–0.1103 Gy) in males and 0.0022 Gy (median 0.0002, min–max 0.00–0.1727 Gy) in females (Tables 1 and 2).

**Table 1:** Distribution of the study cohort workers by cumulative stomach absorbed dose from external gamma rays.

Cumulative stomach gamma-dose, Gy	Males		Females		Both sexes	
	Number	%	Number	%	Number	%
***Entire cohort***						
**[0.0–0.2)**	8410	50.40	3327	58,48	11737	52,45
**[0.2–0.5)**	3494	20.94	923	16,22	4417	19,74
**[0.5–1.0)**	2166	12.98	683	12,01	2849	12,73
**[1.0+**	2618	15.68	756	13,29	3374	15,08
**Total**	16688	100.00	5689	100,00	22377	100,00
**Mean ± SD**	0.48 ± 0.68		0.40 ± 0.59		0.46 ± 0.66	
**Median (Min–Max)**	0.19 (0.0–7.35)		0.13 (0.0–5.63)		0.18 (0.00–7.35)	
***Stomach cancers***						
**[0.0–0.2)**	90	32.14	32	50.79	122	35.57
**[0.2–0.5)**	69	24.64	14	22.22	83	24.20
**[0.5–1.0)**	52	18.57	7	11.11	59	17.20
**[1.0+**	69	24.65	10	15.88	79	23.03
**Total**	280	100.00	63	100.00	343	100.00
**Mean ± SD**	0.70 ± 0.84		0.48 ± 0.83		0.66 ± 0.83	
**Median (Min–Max)**	0.38 (0.00–4.96)		0.19 (0.00–4.28)		0.33 (0.00–4.96)	

**Table 2:** Distribution of the study cohort workers by cumulative stomach absorbed dose from internally deposited alpha-particles of incorporated plutonium.

Cumulative stomach alpha-dose, Gy	Males		Females		Both sexes	
	Number	%	Number	%	Number	%
***Entire cohort***						
**[0–0.0002)**	2930	52.57	1256	51.64	4186	52.29
**[0.0002–0.001)**	1585	28.44	628	25.82	2213	27.64
**[0.001–0.005)**	821	14.73	403	16.57	1224	15.29
**[0.005+**	238	4.26	145	5.97	383	4.78
**Total**	5574	100.00	2432	100.00	8006	100.00
**Mean ± SD**	0.0011 ± 0.0038		0.0022 ± 0.0100		0.0014 ± 0.0064	
**Median (Min–Max)**	0.0002 (0.0–0.1103)		0.0002 (0.0–0.1727)		0.0002 (0.0–0.1727)	
***Stomach cancer cases***						
**[0–0.0002)**	75	47.47	21	46.67	96	47.29
**[0.0002–0.001)**	41	25.95	16	35.56	57	28.08
**[0.001–0.005)**	30	18.99	7	15.56	37	18.23
**[0.005+**	12	7.59	1	2.21	13	6.40
**Total**	158	100.00	45	100.00	203	100.00
**Mean ± SD**	0.0013 ± 0.0029		0.0007 ± 0.0014		0.0012 ± 0.0026	
**Median (Min–Max)**	0.0002 (0.0–0.0173)		0.0002 (0.0–0.0089)		0.0002 (0.0–0.0173)	

Workers were monitored for external radiation exposure since the start of the Mayak PA operation and individual doses from external gamma rays are available for every worker. Internal radiation exposure monitoring system was implemented gradually starting from the end of 1960s, that is why alpha radiation doses were estimated only for 31% of workers who had been exposed to plutonium-239 aerosols during production activities. Doses from exposure to alpha emitters were estimated using measurements of plutonium content in workers’ urine samples. Individuals controlled for plutonium intake during production activities (those for whom regular bioassays were performed to measure plutonium content) are referred to as ‘monitored workers’ and those workers for whom such bioassays were not performed are referred to as ‘non-monitored workers’ [20, 26].

To assess the effect of internal alpha-particle exposure for workers with available bioassay measurements of plutonium alpha activity, in the present study we adapted a surrogate index for internal alpha-dose as suggested in Shilnikova NS et al. [27]. It is based on occupational history information and takes into account a type of occupational activity, period of employment and labor conditions in different calendar periods at different production departments what influenced levels of internal alpha-radiation exposure to plutonium. The surrogate index was used in a series of epidemiological studies of Mayak PA workers [17, 18, 27, 28] and provides estimates for the following categories of workers: 1—reactor plant workers hired between 1948 and 1982; 2 –radiochemical plant workers hired between 1954 and 1982, main plutonium department workers hired between 1964 and 1982 and plutonium auxiliary department workers hired between 1959 and 1982; 3 –plutonium auxiliary department workers hired between 1950 and 1958, radiochemical plant workers hired between 1948 and 1953 and main plutonium department workers hired between 1959 and 1963; 4 –plutonium auxiliary department workers hired between 1948 and 1949 and main plutonium department workers hired 1954–1958; 5 –main plutonium department workers hired between 1950 and 1953; and 6 –main plutonium department workers hired between 1948 and 1949. Since category 5 of the surrogate dose accounted for only one StoCa case, categories 5 and 6 were combined to perform the analysis.

Mayak PA workers could be potentially exposed to X-rays during diagnostic medical procedures. *MWDS-2008* dosimetry system provides estimates for stomach absorbed doses from X-ray examinations. The mean (± standard deviation) X-ray doses over the follow-up period were 0.071 ± 0.093 Gy (min-max 0.0–0.599 Gy) in workers diagnosed with StoCa and 0.024 ± 0.058 Gy (min-max 0.0–0.669 Gy) in StoCa-free workers. These doses from medical exposure were not considered in the risk analysis because they were 10–20 times lower than gamma doses from occupational exposure and were available not for all members of the study cohort (only for 85% of workers with StoCa and for 74% of workers without StoCa).

### Statistical analysis

The statistical analysis of the dataset was conducted using the same methods as in previous studies of the Mayak PA cohort [17, 18, 28]. The Poisson regression was used to test for an association of StoCa incidence with both stomach gamma and alpha doses. For each worker, person-years at risk were taken into account over time from a date of hire at one of the main Mayak PA facilities in 1948–1982 to a date of exit from the study, which was a date of the earliest among the following events: StoCa diagnosis, date of death, date when the last information was reported in medical documents for migrants, or 31.12.2013.

Tabulations of person-years at risk were created with the DATAB module of the EPICURE software [29]. Data were cross-classified by sex, attained age (15 categories by 5-y age intervals: < 20; 20–25,…,80–85, > 80 years), age at hire at main Mayak PA facilities (3 categories: < 20, 20–30, > 30 years), smoking status (4 categories: unknown, nonsmoker, ex-smoker and smoker), type of facility (3 categories: reactor, radiochemical production and plutonium production), alcohol consumption (4 categories: unknown, seldom-drinker, moderate-drinker, heavy-drinker), stomach gamma-dose (9 categories: 0–0.10, > 0.10–0.20, > 0.20–0.50, > 0.50–0.75, > 0.75–1.00, > 1.00–1.50, > 1.50–2.00, > 2.00–3.00, >3.00 Gy) and stomach alpha dose (6 categories: 0.00–0.0001, > 0.0001–0.0002, > 0.0002–0.0005, > 0.0005–0.0010, > 0.0010–0.0020, > 0.0020). To assess a contribution of a latent period in the effect of radiation exposure, an additional analysis considered external gamma and internal alpha doses that were lagged for 0, 5, 10, 15 and 20 years. An additional analysis that took into account smoking index (a number of packs of cigarettes smoked a day multiplied by a number of years during which a person was smoking, pack-y) rather than smoking status was also conducted. The following categories of smoking index were considered in the analysis: non-smokers, > 0–10 pack-y, > 10–20 pack-y, > 20 pack-y, unknown smoking index, unknown smoking status.

The data were fitted using the following model:

$$\lambda_{0}\left(g,a,sm,ac,k_{25},k_{26},k_{29}\right)\cdot \left(1+ERR_{ed,id,sur}\right)$$ (1)

where *λ*_*0*_ is the background cancer incidence rate for radiation-free environment (dose = 0) that depends on attained age (*a*), sex (*g*), smoking status or smoking index (*sm*), alcohol consumption (*ac*), concomitant diseases: *k25* is stomach ulcer, *k26* is duodenal ulcer, *k29* is gastritis and duodenitis; *ERR* is an excess relative risk representing the combination of excess relative risks due to external gamma ray exposure (*ERRed*), internal alpha radiation exposure (*ERRid*) for monitored workers in the model, and categories of the surrogate internal alpha-dose in non-monitored workers of the radiochemical and plutonium production plants (*ERRsur*). More specifically the ERR was modeled as:

$$ERR=ERR_{ed}+ERR_{id}+ERR_{sur}$$ (2)

To control for background factors affecting the StoCa risk, the analyses allowed for various parametric and non-parametric stratification models. Results provided by all these models showed that attained age, sex, smoking status (or smoking index), alcohol consumption and digestive disorders (stomach and duodenal ulcer, gastritis and duodenitis) were the most important factors in modeling the background rates for StoCa incidence. The parametric approach produced a slightly better description of the background rates compared to the non-parametric approach, and this approach was also used in earlier Mayak cohort studies [17, 18, 28].

Finally, the main baseline risk model included a logarithmic function of attained age for a certain sex, squared logarithm of attained age, sex-specific smoking status and alcohol consumption, and took into account digestive disorders (stomach and duodenal ulcer, gastritis and duodenitis) if they had been manifested not later than two years before the exit of a participant out of the study (hereinafter, “SmSta-adj model”). To assess the baseline risk, a model that considered smoking index rather than smoking status was used (hereinafter, “SmInd-adj model”).

A dose-response analysis was based on a linear non-threshold model (LNT, 1 + β_1_D), and additionally it was conducted using non-linear models: quadratic (Q, 1 + β_2_D^2^), linear-quadratic (LQ, 1 + β_1_D + β_2_D^2^), and linear-exponential (LE, 1 + β_1_Dexp(-β_3_D)). The quality of data fit was assessed for non-linear models vs. LNT. Variations in maximum likelihood were used to compare nested models, and the Bayesian information criteria (BIC) was used for non-nested models.

Modifications of the radiogenic risk by the following factors were assessed: attained age, sex, age at first employment at the main facilities, etc. All types of analyses were run using the AMFIT module of the EPICURE software [29]. The maximum likelihood technique was used to assess levels of statistical significance of the results and to estimate bounds of 95% confidence intervals (CIs). All *p*-values quoted were two-sided and a statistical significance level of 5% was used. The assessment was based on Wald’s statistics if a bound of a confidence interval was not defined.

## Results

The study considered 343 cases of StoCa (280 cases in males and 63 in females) diagnosed in workers of the study cohort over a period from a date of hire at the Mayak PA to 31.12.2013. In 248 (72%) workers the diagnosis was confirmed with histological examinations. At a date of the diagnosis, StoCa cases were distributed by a tumor site as follows: cardia StoCa were detected in 54 (16%) workers, non-cardia StoCa were detected in 215 (63%) workers and total StoCa were detected in 74 (21%) workers.

As mentioned above, the best fit of baseline StoCa data was provided by the model that took into account the following parameters: attained age, smoking status, alcohol consumption, stomach and duodenal ulcer, chronic gastritis and duodenitis (SmSta-adj model). The number of StoCa cases in females was relatively modest, that is why it was not possible to assess the association of the disease risk with occupational radiation exposure taking into account non-radiation factors. The analysis of the radiogenic risk in the study cohort was conducted for all workers of the study cohort combined rather than for sex-specific groups, and for male workers separately (these results are summarized in S1–S4 Tables).

Table 3 summarizes results of the analysis of the StoCa incidence risk associated with occupational radiation doses based on the LNT model (both sexes). No association of the StoCa incidence risk with the stomach alpha dose was observed (the ERR estimates for monitored workers and for surrogate dose categories 2–4 were not significant either adjusted or unadjusted for gamma-ray exposure; the ERR resulting from the analysis limited to monitored workers was insignificant either). The significant increase in the ERR of stomach cancer was found only for the combined surrogate dose category (5+6) implying lag periods of 0, 5, 10 and 15 years (Table 3).

**Table 3:** Excess relative risks of stomach cancer incidence in the study cohort associated with external gamma and internal alpha doses (for different lag periods and both sexes, SmSta-adj model).

Estimate		ERR/Gy (95%CI) for various lag periods				
		0 y	5 y	10 y	15 y	20 y
***Model*: *ERR = ERRed + ERRid + ERRsur***						
**ERRed /Gy**		0.17 (-0.02, 0.43)	0.18 (-0.01, 0.45)	0.13 (-0.04, 0.37)	0.13 (-0.05, 2.08)	0.13 (-0.34^W^, 0.60^W^)
ERRid /cGy (males)*		0.36 (-0.69^W^, 1.76)	0.27 (-0.92^W^, 1.87)	0.16 (-0.26, 1.94)	-0.07 (-0.26, 2.08)	-0.16 (-4.09^W^, 3.77^W^)
**ERRsur**	**2**	0.13 (-0.24, 0.64)	0.12 (-0.25, 0.63)	0.10 (-0.26, 0.58)	0.09 (-0.26, 0.57)	0.04 (-0.63^W^, 0.71^W^)
	**3**	0.42 (-0.18, 1.27)	0.43 (-0.17, 1.28)	0.42 (-0.16, 1.24)	0.41 (-0.16, 1.23)	0.22 (-0.85^W^, 1.30^W^)
	4 (males)*	0.30 (-0.47, 1.62)	0.29 (-0.47, 1.61)	0.26 (-0.48, 1.52)	0.24 (-0.48, 1.49)	0.17 (-1.55^W^, 1.89^W^)
	5–6 (males)*	1.64 (0.09, 4.21)	1.63 (0.08, 4.17)	1.61 (0.13, 4.07)	1.60 (0.13, 4.01)	0.77 (-1.92^W^, 3.47^W^)
***Model*: *ERR = ERRed***						
**ERRed /Gy**		0.19 (0.01, 0.44)	0.20 (0.01, 0.45)	0.13 (-0.04, 0.36)	0.12 (-0.05, 0.34)	0.08 (-0.07, 0.31)
***Model*: *ERR = ERRid + ERRsur***						
**ERRid /сGy**		0.18 (-0.27, 1.13)	0.11 (-0.27, 1.18)	-0.02 (-0.28, 1.19)	-0.13 (-0.67^W^, 0.42^W^)	-0.14 (-1.13^W^, 0.85^W^)
**ERRsur**	**2**	0.07 (-0.27, 0.52)	0.07 (-0.27, 0.51)	0.06 (-0.28, 0.50)	0.06 (-0.32^W^, 0.44^W^)	0.05 (-0.33^W^, 0.43^W^)
	**3**	0.38 (-0.17, 1.15)	0.37 (-0.17, 1.14)	0.37 (-0.17, 1.12)	0.37 (-0.27^W^, 1.00^W^)	0.43 (-0.22^W^, 1.09^W^)
	4 (males)*	0.20 (-0.49, 1.37)	0.20 (-0.49, 1.36)	0.19 (-0.49, 1.35)	0.19 (-0.70^W^, 1.09^W^)	0.29 (-0.62^W^, 1.20^W^)
	5–6 (males)*	1.55 (0.15, 3.84)	1.54 (0.15, 3.82)	1.53 (0.14, 3.79)	1.52 (-0.26^W^, 3.29^W^)	1.60 (-0.18^W^, 3.38^W^)
***Model*: *ERR = ERRid***						
**ERRid /сGy**		0.16 (-0.44^W^, 1.10)	0.09 (-0.56^W^, 1.14)	-0.04 (-0.09, 1.15)	-0.12 (-0.74^W^, 0.50^W^)	-0.16 (-0.86^W^, 0.55^W^)

ERRed/Gy denotes an excess relative risk per 1 Gy of stomach absorbed dose from external gamma rays;

ERRid/cGy denotes an excess relative risk per 1 cGy of absorbed dose from internal alpha particles for monitored workers;

ERRsur denotes an excess relative risk in categories of surrogate dose for non-monitored workers;

^W^ denotes that an estimate was based on Wald’s statistics if an bound of a confidence interval was not defined;

* denotes that the category included only male workers.

The significant linear association of the StoCa incidence risk in the study cohort was observed with the stomach gamma dose (unadjusted for internal alpha exposure): ERRed/Gy = 0.19 (95% CI: 0.01, 0.44) with the 0 y lag implied and ERRed/Gy = 0.20 (95% CI: 0.01, 0.44) with the 5 y lag implied (Table 3). The male-restricted analysis provided similar results, however, the ERRed/Gy estimates were modestly higher: ERRed/Gy = 0.21 (95% CI: 0.01, 0.50) with the 0 y lag implied and ERRed/Gy = 0.22 (95% CI: 0.01, 0.51) with the 5 y lag implied (S1 Table). ERRed/Gy estimates with 10, 15 and 20 y lags implied were not significant (Tables 3 and S1).

We did not observe any modifications of the ERR for external gamma-ray exposure by sex, attained age, smoking status, alcohol consumption, stomach and duodenal ulcer, chronic gastritis and duodenitis (Table 4). Neither the ERRed estimate was significantly modified by other occupation-related factors, such as a type of facility, age and period of hire or duration of employment (Table 5). The assessment of radiogenic risk modifications considering only male workers provided similar results (S2 and S3 Tables).

**Table 4:** Modification of the excess relative risk of stomach cancer in the study cohort associated with external gamma ray exposure by non-radiation factors (both sexes, SmSta-adj model).

Factors		Number of cases	ERRed/Gy	
			Unadjusted for internal alpha-radiation exposure	Adjusted for internal alpha-radiation exposure
**Sex**	**Males**	280	0.21 (0.01, 0.50)	0.19 (-0.02, 0.49)
	**Females**	63	0.10 (-0.38^W^, 0.74)	0.09 (-0.39^W^, 0.74)
	*p* value (test for heterogeneity)		> 0.50	> 0.50
**Age**	**< 50**	78	0.36 (-0.05, 1.14)	0.26 (-0.12, 1.03)
	**50–60**	89	0.76 (0.17, 1.90)	0.80 (0.17, 2.04)
	**60–70**	89	0.12 (-0.13, 0.57)	0.09 (-0.16, 0.57)
	**70+**	87	-0.06 (-0.32^W^, 0.24)	-0.07 (-0.35^W^, 0.26)
	*p* value (test for heterogeneity)		0.069	0.084
	*p* value (trend)		0.143	0.248
**Smoking**	**Non-smokers**	111	0.07 (-0.28^W^, 0.49)	0.08 (-0.29^W^, 0.52)
	**Former smokers**	67	0.01 (-0.29^W^, 0.43)	-0.06 (-0.35^W^, 0.38)
	**Smokers**	159	0.45 (0.11, 1.00)	0.44 (0.08, 1.02)
	**Unknown**	6	1.49 (-3.32^W^, 27.99)	0.75 (-2.94^W^, 25.02)
	*p* value (heterogeneity test)		0.280	0.282
**Alcohol consumption**	**Non-drinkers**	65	0.24 (-0.32^W^, 1.01)	0.24 (-0.33^W^, 1.03)
	**Moderate drinkers**	153	0.17 (-0.08, 0.57)	0.15 (-0.10, 0.55)
	**Heavy drinkers**	89	0.13 (-0.09, 0.53)	0.11 (-0.14, 0.53)
	**Unknown**	36	0.50 (-0.57^W^, 2.80)	0.59 (-0.62^W^, 3.31)
	*p* value (heterogeneity test)		> 0.50	> 0.50
**Stomach ulcer**	**No**	312	0.19 (0.01, 0.45)	0.18 (-0.02, 0.46)
	**Yes**	31	0.19 (-0.48^W^, 1.38)	0.14 (-0.50^W^, 1.20)
	*p* value (heterogeneity test)		> 0.50	> 0.50
**Duodenal ulcer**	**No**	326	0.20 (0.01, 0.45)	0.21 (0.01, 0.49)
	**Yes**	17	0.16 (-0.67^W^, 2.66)	-0.10 (-0.57^W^, 0.70^W^)
	*p* value (heterogeneity test)		> 0.50	0.345
**Gastritis and duodenitis**	**No**	149	0.23 (-0.03, 0.64)	0.24 (-0.05, 0.70)
	**Yes**	194	0.17 (-0.05, 0.49)	0.13 (-0.08, 0.46)
	*p* value (heterogeneity test)		> 0.50	> 0.50

^W^ denotes that an estimate was based on Wald’s statistics if a bound of a confidence interval was not defined.

**Table 5:** Modification of the excess relative risk of stomach cancer associated with external gamma ray exposure in the study cohort by occupation-related factors (both sexes, SmSta-adj model).

Factors		Number of cases	ERRed/Gy	
			Unadjusted for internal alpha-radiation exposure	Adjusted for internal alpha-radiation exposure
**Type of facility**	**Reactors**	87	0.30 (-0.09, 1.16)	0.27 (-0.11, 1.09)
	**Radiochemical plant**	136	0.18 (-0.04, 0.55)	0.17 (-0.07, 0.57)
	**Plutonium production plant**	120	0.60 (0.03, 1.57)	0.47 (-0.13, 1.51)
	*p* value (test for heterogeneity)		> 0.50	> 0.50
**Age at first employment**	**< 20**	58	-0.02 (-0.41^W^, 0.51)	-0.01 (-0.43^W^, 0.54)
	**20–30**	162	0.20 (-0.04, 0.58)	0.18 (-0.06, 0.56)
	**30+**	123	0.31 (-0.01, 0.88)	0.30 (-0.07, 0.93)
	*p* value (test for heterogeneity)		> 0.50	> 0.50
**Period of employment**	**1948–1958**	208	0.16 (-0.03, 0.45)	0.16 (-0.04, 0.48)
	**1959–1972**	107	0.13 (-0.76^W^, 1.61)	0.05 (-0.82^W^, 1.52)
	**1973–1982**	28	-0.22 (-4.49^W^, 11.91)	-0.30 (-4.59^W^, 12.36)
	*p* value (test for heterogeneity)		> 0.50	> 0.50
**Duration of employment**	**< 10**	83	0.10 (-0.23^W^, 0.57)	0.11 (-0.27^W^, 0.64)
	**10+**	260	0.24 (0.03, 0.56)	0.21 (-0.01, 0.53)
	*p* value (test for heterogeneity)		> 0.50	> 0.50

^W^ denotes that an estimate was based on Wald’s statistics if a bound of a confidence interval was not defined.

**Fig 1:**
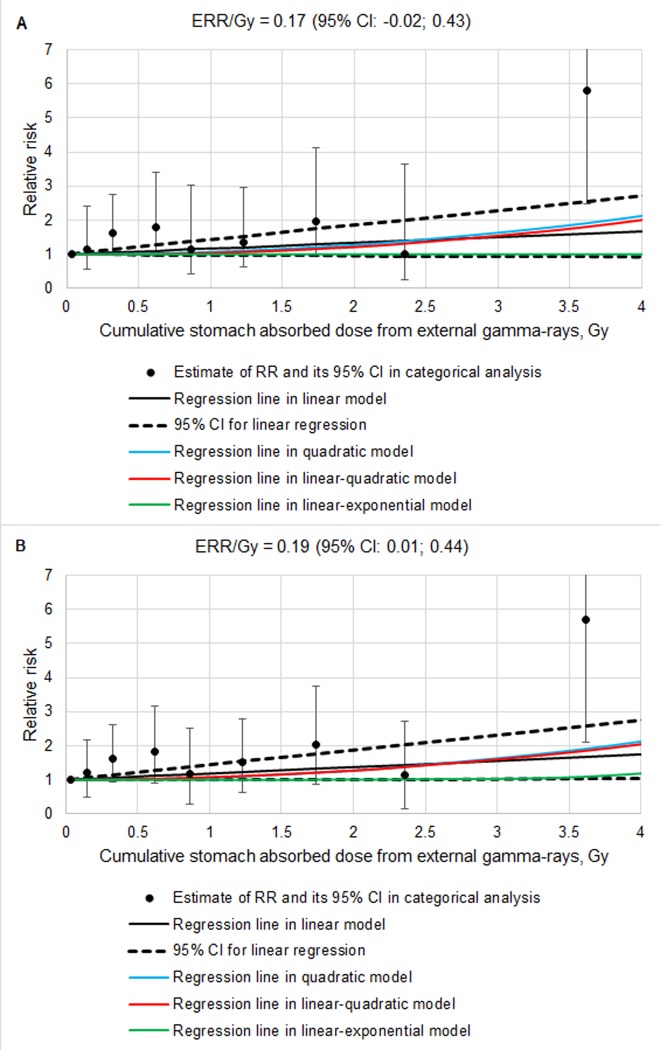
The association of stomach cancer incidence in the study cohort with dose from external gamma rays (linear and non-linear SmSta-adj models, both sexes). A − Adjusted for internal radiation dose, B − Unadjusted for internal radiation dose.

Results of the analysis of the StoCa incidence risk associated with the stomach gamma-dose (SmSta-adj model) are demonstrated in Fig 1 (for both sexes) and in S1 Fig (only for males). The dose-response analysis for StoCa incidence risk in the study cohort associated with stomach gamma doses did not reveal any advantages of non-linear models versus the LNT model, neither it revealed any significant differences between non-linear and LNT models (Table 6 summarizes results for both sexes, S4 Table–for male workers separately). Only when the adjustment for internal alpha radiation was included and the analyses considered both male and female workers, differences between LNT and LE models were significant (*p*-value = 0.048) but the linear parameter of the LE model still remained insignificant (Table 6).

**Table 6:** The association of stomach cancer incidence in the study cohort with dose from external gamma rays (linear and non-linear SmSta-adj models, both sexes).

Model	Model parameters			Deviation	Number of parameters	Records used	Criteria for comparison
	*β*_*1*_	*β*_*2*_	*β*_*3*_				
**Unadjusted for internal radiation dose**							
Linear	0.19 (0.01, 0.44)	−	−	5419.853	14	740272	−
Quadratic	−	0.07 (0.01, 0.16)	−	5419.163	14	740272	*ΔBIC* = 0.690
Linear-quadratic	0.02 (-0.35, 0.50)	0.06 (-0.11^W^, 0.21)	−	5419.148	15	740272	*p* value = 0.401
Linear-exponential	0.01 (-2.62^W^, 2.65^W^)	−	-2.70 (-320.80^W^, 315.40^W^)	5430.195	15	740272	*p* value = na
**Adjusted for internal radiation dose**							
Linear	0.17 (-0.02, 0.43)	−	−	5413.261	19	740272	−
Quadratic	−	0.07 (+0.00, 0.16)	−	5412.316	19	740272	*ΔBIC* = 0.945
Linear-quadratic	-0.03 (-0.41, 0.46)	0.07 (-0,10^W^, 0.23)	−	5412.300	20	740272	*p* value = 0.327
Linear-exponential	+0.00 (-0.02^W^, 0.20)	–	1.19 (0.01, 2.89)	5409.353	20	740272	*p* value = 0.048

^W^ denotes that an estimate was based on Wald’s statistics if a bound of a confidence interval was not defined.

Results of the dose-response analysis for StoCa incidence risk in the study cohort that were based on LNT SmInd-adj model, are presented in S5 and S6 Tables. The significant increase in the ERR of stomach cancer was found only for the combined surrogate alpha-dose category (5+6) implying lag periods of 0, 5, 10, 15 years. No association was found for the StoCa incidence risk with the stomach gamma and alpha doses (S5 and S6 Tables).

In general, the ERRs based on LNT SmSta-adj and LNT SmInd-adj models were similar (Tables 3 and S5 for all workers, S1 and S6 Tables only for males). However, with smoking index rather than smoking status taken into account (LNT SmInd-adj model) ERRs/Gy of the stomach gamma dose (unadjusted for internal alpha exposure) did not reach statistical significance: ERRed/Gy = 0.17 (95% CI: -0.00, 0.40) with the 0 y lag implied and ERRed/Gy = 0.17 (95% CI: -0.00, 0.41) with the 5 y lag implied (S5 Table for both sexes) and ERRed/Gy = 0.17 (95% CI: -0.00, 0.44) with the 0 y lag implied and ERRed/Gy = 0.18 (95% CI: -0.01, 0.45) with the 5 y lag implied (S6 Table only for males).

## Discussion

Results of studies of solid cancer incidence (1948–2004 follow-up period) [17] and mortality (1948–2008 follow-up period) [18] in the Mayak PA worker cohort were published earlier and provided risk estimates for StoCa among other outcomes. These results were based on gamma and plutonium alpha doses from external and internal exposures, respectively. Gamma doses were provided by the *MWDS-2008*. For this study, the cohort follow-up was extended to 31.12.2013 and included 564,664 person-years, hence the number of considered StoCa cases increased up to 343. Improved internal plutonium-dose estimates were provided by the *MWDS-2013* dosimetry system for the present analyses.

Main approaches used to run statistical analyses in this study were similar to those used earlier by Hunter et al. [17] and Sokolnikov et al. [18]. An important difference is that in this study the baseline risk model included alcohol consumption and digestive diseases (peptic ulcer, gastritis and duodenitis) that could be induced by *H*. *pylori* infection and contribute in StoCa occurrence [6, 25] in addition to sex, attained age and smoking status variables.

The present study demonstrated the significant linear association of the StoCa incidence risk with the gamma-ray dose from external exposure in workers first employed at the Mayak PA in 1948–1982 (0 y and 5 y lag periods, smoking status as a confounder): the ERRed/Gy estimate was modestly higher than estimates resulting from the incidence analysis performed by Hunter et al. (ERRed/cSv = 0.15 (95% CI: -0.01, 0.39); p<0.07) and the mortality analysis performed by Sokolnikov et al. (ERRed/Gy = 0.12 (95% CI: -0.03, 0.31); p<0.06) also based on LNT models [17, 18]. It should be noted that the study by Sokolnikov et al. in addition to workers of main facilities of the Mayak PA considered also employees of auxiliary facilities (mechanical repair plant and water-treatment departments) [18].

The analysis of the radiogenic risk of the StoCa incidence in this study was also conducted considering smoking index as a confounding factor, but the resulting ERRs/Gy of stomach gamma dose did not reach statistical significance (*p-*value > 0.1). As noted above, this might be attributed to a smaller number of the study cohort members with available complete quantitative data on smoking compared to the number of workers with available information on smoking status.

Most of the studies considering nuclear energy and atomic industry workers from different countries, medical personnel exposed to ionizing radiation at work and air crew members did not find any significant effect of external low-LET radiation on StoCa risks [14–16, 30–34]. A solid cancer mortality analysis of French, UK and USA nuclear workers that included 308,297 individuals followed up over 8.2 mln person-years did not find a significant association of StoCa with external gamma-ray exposure (mean cumulative stomach absorbed doses were 22.8 mGy in males and 4.9 mGy in females) based on maximum likelihood Poisson regression. However, the use of a hierarchical Poisson regression with Markov Chain Monte Carlo resulted in ERRed/Gy of 0.88 (90% CI: 0.01, 1.82) (10 y lag implied) [14]. A meta-analysis of studies considering nuclear workers occupationally exposed to low levels of radiation revealed a significant decrease in StoCa mortality compared to background population rates [35].

A significant association of the StoCa risk with acute gamma-neutron exposure was shown in the Life Span Study (LSS) of the Japanese atomic-bomb survivors, but reported ERR/Gy estimates were higher than those in the Mayak PA workers: a mortality risk analysis provided the sex and age-averaged ERR/Gy estimate for StoCa of 0.28 (95% CI: 0.14, 0.42) [8]. In the LSS cohort the sex-averaged ERRs/Gy based on the LNT model for 70 years old individuals exposed at the age of 30 years were 0.33 (95% CI: 0.17, 0.52) with mortality considered as an outcome [8] and 0.34 (90% CI: 0.22, 0.47) with incidence of StoCa considered as an outcome [7]. The study by Sakata R. et al. (2019) that considered an extended follow-up of the LSS cohort (1958–2009) reported StoCa incidence sex-averaged ERR/Gy at age 70 of 0.33 (95% CI: 0.20, 0.47); this estimate took into account a multiplicative joint effect with smoking history [36].

Similarly to the majority of studies of occupational radiation exposure effects and the LSS cohort studies, the radiogenic risk estimates were based on the LNT model implying a 10-y lag and taking into account a background risk associated with sex, age and other non-radiation factors. Usually, smoking, alcohol consumption and for StoCa *H*.*pylori* infection and related diseases were not taken into account. In the present study as well as in earlier studies [17, 18] analyses of the StoCa risk associated with external gamma-ray exposure did not demonstrate non-linearity of the ERR/Gy association, in addition, in the present study significant estimates were observed only when 0 and 5 year lags were implied.

Radiogenic risk estimates reported for the LSS cohort are a gold standard that radiation safety regulations are based on. However, discussions on whether it is appropriate to use them for approximation to other populations, especially in case of StoCa for which higher incidence rates are reported in Japan compared to other countries [7, 37], still continue as well as discussions on potential implications of these estimates for limitation of occupational low-dose radiation exposure. Other approaches to analyzing accumulated data aimed to update dose-response relationships and to estimating radiogenic risks considering uncertainties are applied. For instance, a mortality analysis for StoCa performed using simulation techniques and limited to the LSS cohort members exposed at stomach absorbed doses from gamma rays and neutrons ranged between 0–20 and 5–500 mSv, respectively, demonstrated a non-linear dose association with the optimal latent period of 11.89 years [38, 39]. Molecular mechanisms of StoCa following the atomic bomb radiation exposure are investigated [40–41].

The increased stomach cancer risk was found in patients following radiation therapy of Hodgkin’s lymphoma, testicles and cervix cancer, peptic ulcer [9–13], however, the estimates were highly varying. The excess odds ratio (EOR)/Gy for StoCa considering a pooled dataset that included three case-control studies of patients following therapeutic radiation therapy of Hodgkin’s lymphoma, testicles and cervix cancer was 0.091 (95% CI: 0.036, 0.20) [9]. The EOR/Gy estimate increased with time after exposure, and 20 years later it was 0.38 (95%CI: 0.12, 1.04) [9]. No effect of radiotherapy for benign gynecologic disorders was observed on the StoCa mortality risk [42]. ERR/Gy estimates for StoCa following therapeutic radiation exposure for peptic ulcer reported in different studies were of the similar levels: 0.06 (95%CI: 0.02, 0.1 0) 10+ years after exposure [43] and 0.042 (95%CI: -0.002, 0.119), *p*<0.07 [13].

No associations of risks of StoCa with internal plutonium exposure were observed in the Mayak PA worker cohort either in this or previous studies [17, 18]. Other cohorts of workers exposed to plutonium and uranium demonstrated similar results [44–47]. The performed analysis revealed the significant increase in the StoCa risk in the combined category (5+6) of the surrogate alpha dose. This combined category included non-monitored workers involved in production activities at the main plutonium production plants of the Mayak PA during the period of setting up of technology procedures when concentrations of plutonium aerosols and other chemical agents in the air in production rooms were the highest. An additional investigation and discussion are needed to explain potential reasons of the increased StoCa risk in these workers.

A distinguishing feature of this study is the considerable number of non-radiation factors taken into account demonstrating the significant effect on the baseline risk in the Mayak PA cohort. According to available estimates, 78.5% of the Russian population is infected with *H*. *pylori* [24], that is why the baseline risk model included digestive diseases that could be potentially induced by this pathogen. Within the present study no modification of radiogenic StoCa risk by sex, attained age, age at first employment at the Mayak PA, duration of employment and type of facility, smoking status, alcohol consumption and concomitant digestive diseases was revealed. The LSS study showed that the age at exposure had a considerable effect on the risk of malignant neoplasms, including StoCa [7, 8, 36]. Additionally, some site-specific studies give evidence to the effect of smoking and diet on the radiogenic StoCa risk in atomic bomb survivors [48, 49].

It should be noted that the study was performed retrospectively and the analyses were based on data reported in medical records of workers over the whole follow-up period. Invasive examinations of the gastrointestinal tract and laboratory tests were carried out for workers only when it was medically necessary. Diagnostics tools that were used in clinical practice for digestive disorders were changing during 1948–2013. So, endoscopy was implemented only in the end of 1970s, *H*. *pylori* infection tests have been used only in the recent decades.

Unavailable for workers of the study cohort information on such considerable risk factors for StoCa, as *H*. *pylori* infection and nitrite and nitrate intakes with drinking water and food [50], potentially might confound the results obtained in the present analysis. Additionally, a relatively low number of StoCa cases did not allow for a more detailed analysis of the modification effect of quantitative smoking parameters on the StoCa incidence risk. It should be specifically highlighted that the association of the StoCa incidence risk with the stomach gamma-dose was significant when smoking status was taken into account as a confounder. When the ERR/Gy of external gamma-exposure was adjusted for smoking index, the risk estimate failed to reach statistical significance.

This resulted in a number of limitations of the study: the contribution of *H*. *pylori* infection was not evaluated; cases of atrophic gastritis known to be a considerable risk factor for StoCa were not categorized separately; dietary habits of workers were not taken into account, neither were occupational and environmental exposures to chemicals. It is known that cardia StoCa and non cardia StoCa are etiologically different [2]. However, the analysis performed in this study did not take into account a site of tumor location within a stomach, because the number of cardia StoCa revealed in workers of the study cohort was small.

## Conclusion

The analysis considering the cohort of Mayak PA workers first employed at the main facilities in 1948–1982 demonstrated the significant linear association of the StoCa incidence risk with the cumulative stomach absorbed gamma-ray dose from external exposure. The ERRed/Gy estimated relative to the background risk taking into account sex, attained age, smoking status, alcohol consumption and chronic digestive diseases (stomach and duodenal ulcer, gastritis and duodenitis) was comparable to estimates provided by previous studies of the cohort. No modification of the StoCa risk association with external gamma ray exposure by non-radiation factors was observed. No association was found for the stomach cancer incidence risk with exposure to internally deposited alpha particles from incorporated plutonium.

## Supporting information

S1 Table Excess relative risks of stomach cancer incidence risk in the cohort of Mayak PA workers associated with doses from external gamma rays and internally deposited alpha particles (for different lag periods, males, SmSta-adj model). ERRed/Gy denotes an excess relative risk per 1 Gy of stomach absorbed dose from external gamma rays; ERRid /сGy denotes excess relative risk per 1 cGy of the stomach absorbed internal alpha radiation dose for monitored workers; ERRsur denotes excess relative risks in categories of surrogate doses for non-monitored workers; ^W^ denotes that an estimate was based on Wald’s statistics if a bound of a confidence interval was not defined.
(DOCX)

S2 Table Modifications of excess relative risks of stomach cancer incidence in the study cohort associated with external gamma ray exposure by non-radiation factors (males, SmSta-adj model). ^W^ denotes that an estimate was based on Wald’s statistics if a bound of a confidence interval was not defined.
(DOCX)

S3 Table Modifications of the excess relative risks of stomach cancer in the study cohort associated with external gamma rays by occupational factors (males, SmSta-adj model). ^W^ denotes that an estimate was based on Wald’s statistics if a bound of a confidence interval was not defined.
(DOCX)

S4 Table The association of stomach cancer incidence risk in the study cohort with dose from external gamma rays (linear and non-liner models, males, SmSta-adj model). ^W^ denotes that an estimate was based on Wald’s statistics if a bound of a confidence interval was not defined.
(DOCX)

S5 Table Excess relative risks of stomach cancer incidence in the study cohort associated with external gamma and internal alpha doses (for different lag periods and both sexes, SmInd-adj model). ERRed/Gy denotes an excess relative risk per 1 Gy of stomach absorbed dose from external gamma rays; ERRid/cGy denotes an excess relative risk per 1 cGy of absorbed dose from internal alpha particles for monitored workers; ERRsur denotes an excess relative risk in categories of surrogate dose for non-monitored workers;^W^ denotes that an estimate was based on Wald’s statistics if an bound of a confidence interval was not defined; * denotes that the category included only male workers.
(DOCX)

S6 Table Excess relative risks of stomach cancer incidence risk in the cohort of Mayak PA workers associated with doses from external gamma rays and internally deposited alpha particles (for different lag periods, males, SmInd-adj model). ERRed/Gy denotes an excess relative risk per 1 Gy of stomach absorbed dose from external gamma rays; ERRid /сGy denotes excess relative risk per 1 cGy of the stomach absorbed internal alpha radiation dose for monitored workers; ERRsur denotes excess relative risks in categories of surrogate doses for non-monitored workers; ^W^ denotes that an estimate was based on Wald’s statistics if a bound of a confidence interval was not defined.
(DOCX)

S1 Fig The association of stomach cancer incidence in the study cohort with dose from external gamma rays (linear and non-linear models SmSta-adj, only males considered). A − Adjusted for internal radiation dose, B − Unadjusted for internal radiation dose.
(DOCX)
