# Supplementary material for: Risk of stomach cancer incidence in a cohort of Mayak PA workers occupationally exposed to ionizing radiation
Source: PLoS One. 2020 Apr 15;15(4):e0231531. doi: 10.1371/journal.pone.0231531 (PMC7159243; doi:10.1371/journal.pone.0231531)
Supplement: S1 Table — ERRed/Gy denotes an excess relative risk per 1 Gy of stomach absorbed dose from external gamma rays; ERRid /сGy denotes excess relative risk per 1 cGy of the stomach absorbed internal alpha radiation dose for monitored workers; ERRsur denotes excess relative risks in categories of surrogate doses for non-monitored workers; W denotes that an estimate was based on Wald’s statistics if a bound of a confidence interval was not defined. (DOCX) [file pone.0231531.s001.docx]

| Table S1 Excess relative risks of stomach cancer incidence risk in the cohort of Mayak PA workers associated with doses from external gamma rays and internally deposited alpha particles (for different lag periods, males, **SmSta-adj model**) | | | | | | | | | | | | |
| --- | --- | --- | --- | --- | --- | --- | --- | --- | --- | --- | --- | --- |
| Estimate | | | ERR/Gy (95%CI) for different lag periods | | | | | | | | | |
|  |  |  | 0 y | | 5 y | 10 y | 15 y | | | 20 y | | |
| *Model: ERR=ERRed+ERRid+ERRsur* | | | | | | | | | | | | |
| ERRed /Gy | | | 0.19 (-0.02, 0.49) | 0.18 (-0.01, 0.45) | | 0.14 (-0.05, 0.43) | 0.14 (-0.05, 2.12) | | | 0.15 (-0.40^W^, 0.69^W^) | | |
| ERRid /cGyр | | | 0.36 (-0.72^W^, 1.80) | 0.27 (-0.96^W^, 1.91) | | 0.17 (-0.24, 1.98) | -0.07 (-0.24, 2.12) | | | -0.19 (-4.11^W^, 3.73^W^) | | |
| ERRsur | 2 | | 0.21 (-0.22, 0.80) | 0.20 (-0.22, 0.79) | | 0.17 (-0.24, 0.73) | 0.16 (-0.24, 0.71) | | | 0.08 (-0.69^W^, 0.84^W^) | | |
|  | 3 | | 0.50 (-0.18, 1.50) | 0.51 (-0.17, 1.52) | | 0.49 (-0.16, 1.45) | 0.48 (-0.16, 1.43) | | | 0.28 (-1.00^W^, 1.56^W^) | | |
|  | 4 | | 0.33 (-0.46, 1.69) | 0.32 (-0.46, 1.68) | | 0.28 (-0.47, 1.57) | 0.26 (-0.48, 1.53) | | | 0.18 (-1.56^W^, 1.92^W^) | | |
|  | 5–6 | | 1.69 (0.10, 4.33) | 1.67 (0.08, 4.29) | | 1.65 (0.14, 4.15) | 1.63 (0.14, 4.10) | | | 0.79 (-1.93^W^, 3.51^W^) | | |
| *Model: ERR=ERRed* | | | | | | | | | | | | |
| ERRed /Gy | | | 0.21 (0.01, 0.50) | 0.22 (0.01, 0.51) | | 0.13 (-0.05, 0.39) | 0.12 (-0.06, 0.37) | | | 0.08 (-0.09, 0.33) | | |
| *Model: ERR= ERRid+ERRsur* | | | | | | | | | | | | |
| ERRid /сGy | | | 0.59 (-0.40^W^, 1.93) | 0.53 (-0.58^W^, 2.05) | | 0.40 (-0.85^W^, 2.12) | | 0.23 (-0.26, 2.31) | | | -0.21 (-1.47^W^, 2.30) | |
| ERRsur | | 2 | 0.15 (-0.24, 0.67) | 0.14 (-0.25, 0.66) | | 0.13 (-0.25, 0.64) | | 0.12 (-0.26, 0.63) | | | 0.11 (-0.32, 0.62) | |
|  |  | 3 | 0.45 (-0.17, 1.34) | 0.44 (-0.17, 1.33) | | 0.42 (-0.18, 1.30) | | 0.41 (-0.19, 1.29) | | | 0.40 (-0.49, 1.26) | |
|  |  | 4 | 0.24 (-0.48, 1.45) | 0.23 (-0.48, 1.43) | | 0.22 (-0.48, 1.40) | | 0.21 (-0.49, 1.39) | | | 0.20 (-0.49, 1.36) | |
|  |  | 5–6 | 1.62 (0.18, 3.99) | 1.61 (0.18, 3.95) | | 1.58 (0.16, 3.90) | | 1.56 (0.16, 3.87) | | | 1.54 (0.16, 3.82) | |
| *Model: ERR= ERRid* | | | | | | | | | | | | |
| ERR_intern_/сGy | | | 0.56 (-0.42^W^, 1.89) | 0.50 (-0.60^W^, 2.01) | | 0.36 (-0.87^W^, 2.07) | | | 0.19 (-1.20^W^, 2.26) | | | -0.22 (-1.09^W^, 2.23) |
| Notes: ERRed/Gy denotes an excess relative risk per 1 Gy of stomach absorbed dose from external gamma rays;  ERRid /сGy denotes excess relative risk per 1 cGy of the stomach absorbed internal alpha radiation dose for monitored workers;  ERRsur denotes excess relative risks in categories of surrogate doses for non-monitored workers;  ^W^ denotes that an estimate was based on Wald’s statistics if a bound of a confidence interval was not defined. | | | | | | | | | | | | |
