# Supplementary material for: Risk of stomach cancer incidence in a cohort of Mayak PA workers occupationally exposed to ionizing radiation
Source: PLoS One. 2020 Apr 15;15(4):e0231531. doi: 10.1371/journal.pone.0231531 (PMC7159243; doi:10.1371/journal.pone.0231531)
Supplement: S2 Table — W denotes that an estimate was based on Wald’s statistics if a bound of a confidence interval was not defined. (DOCX) [file pone.0231531.s002.docx]

| S2 Table. Modifications of excess relative risks of stomach cancer incidence in the study cohort associated with external gamma ray exposure by non-radiation factors (males, **SmSta-adj model**) | | | | |
| --- | --- | --- | --- | --- |
| Factors | | Number of cases | ERRed/Gy | |
|  |  |  | Unadjusted for internal radiation exposure | Adjusted for internal radiation exposure |
| Age | < 50 | 71 | 0.43 (-0.03, 1.34) | 0.34 (-0.09, 1.27) |
|  | 50-60 | 69 | 0.80 (0.16, 2.17) | 0.89 (0.18, 2.44) |
|  | 60-70 | 76 | 0.04 (-0.30^W^, 0.51) | +0.00 (-0.35^W^, 0.50) |
|  | 70+ | 64 | -0.04 (-0.34^W^, 0.34) | -0.05 (-0.37^W^, 0.38) |
|  | *p* value (test for heterogeneity) | | 0.089 | 0.091 |
|  | *p* value (trend) | | 0.146 | 0.241 |
| Smoking | Non-smokers | 50 | 0.01 (-0.45^W^, 0.47^W^) | 0.02 (-0.49^W^, 0.78) |
|  | Former smokers | 67 | +0.00 (-0.29^W^, 0.24) | -0.06 (-0.35^W^, 0.39) |
|  | Smokers | 158 | 0.47 (0.04^W^, 1.03) | 0.47 (0.10, 1.10) |
|  | Unknown | 5 | -0.37 (-1.30^W^, 12.72) | -0.36 (-1.43^W^, 12.67) |
|  | *p* value (test for heterogeneity) | | 0.274 | 0.245 |
| Alcohol consumption | Non-drinkers | 17 | 0.69 (-1.10^W^, 5.94) | 0.75 (-1.16^W^, 6.28) |
|  | Moderate drinkers | 146 | 0.21 (-0.06, 0.64) | 0.19 (-0.08, 0.62) |
|  | Heavy drinkers | 87 | 0.15 (-0.09, 0.58) | 0.14 (-0.12, 0.61) |
|  | Unknown | 30 | 0.47 (-0.65^W^, 2.83) | 0.63 (-0.72^W^, 3.60) |
|  | *p* value (test for heterogeneity) | | > 0.50 | > 0.50 |
| Stomach ulcer | No | 255 | 0.21 (+0.00, 0.51) | 0.20 (-0.02, 0.54) |
|  | Yes | 25 | 0.24 (-0.53^W^, 1.77) | 0.17 (-0.55^W^, 1.47) |
|  | *p* value (test for heterogeneity) | | > 0.50 | > 0.50 |
| Duodenal ulcer | No | 263 | 0.21 (0.01, 0.51) | 0.20 (-0.02, 0.53) |
|  | Yes | 17 | 0.16 (-0.68^W^, 2.67) | 0.14 (-0.70^W^, 2.45) |
|  | *p* value (test for heterogeneity) | | > 0.50 | > 0.50 |
| Gastritis and duodenitis | No | 255 | 0.28 (-0.01, 0.75) | 0.30 (-0.02, 0.83) |
|  | Yes | 25 | 0.16 (-0.08, 0.54) | 0.12 (-0.12, 0.51) |
|  | *p* value (test for heterogeneity) | | > 0.50 | 0.462 |
| Notes: ^W^ denotes that an estimate was based on Wald’s statistics if a bound of a confidence interval was not defined | | | | |
