# Supplementary material for: Risk of stomach cancer incidence in a cohort of Mayak PA workers occupationally exposed to ionizing radiation
Source: PLoS One. 2020 Apr 15;15(4):e0231531. doi: 10.1371/journal.pone.0231531 (PMC7159243; doi:10.1371/journal.pone.0231531)
Supplement: S3 Table — W denotes that an estimate was based on Wald’s statistics if a bound of a confidence interval was not defined. (DOCX) [file pone.0231531.s003.docx]

| Table S3 Modifications of the excess relative risks of stomach cancer in the study cohort associated with external gamma rays by occupational factors (males, SmSta-adj model) | | | | |
| --- | --- | --- | --- | --- |
| Factors | | Number of cases | ERRed/Gy | |
|  |  |  | Unadjusted for internal radiation exposure | Adjusted for internal radiation exposure |
| Type of facility | Reactors | 77 | 0.33 (-0.08, 1.27) | 0.30 (-0.10, 1.18) |
|  | Radiochemical plant | 105 | 0.19 (-0.05, 0.64) | 0.20 (-0.09, 0.70) |
|  | Plutonium plant | 98 | 0.87 (0.15, 2.16) | 0.78 (-0.00, 2.24) |
|  | *p* value | | 0.298 | 0.484 |
| Age at exposure | < 20 | 48 | -0.11 (-0.04, 0.55) | -0.10 (-0.06, 0.59) |
|  | 20 – 30 | 131 | 0.22 (-0.04, 0.66) | 0.21 (-0.06, 0.66) |
|  | 30+ | 101 | 0.34 (-0.00, 0.94) | 0.34 (-0.05, 1.03) |
|  | *p* value | | 0.424 | 0.480 |
| Age at first employment | 1948 – 1958 | 168 | 0.18 (-0.03, 0.52) | 0.19 (-0.04, 0.60) |
|  | 1959 – 1972 | 91 | 0.11 (-0.78^W^, 1.63) | 0.03 (-0.85^W^, 1.53) |
|  | 1973 – 1982 | 21 | 0.32 (-4.90^W^, 17.07) | 0.15 (-5.03^W^, 17.57) |
|  | *p* value | | > 0.50 | > 0.50 |
| Duration of employment | < 10 | 66 | -0.02 (-0.32^W^, 0.45) | -0.03 (-0.38^W^, 0.51) |
|  | 10+ | 214 | 0.31 (0.06, 0.70) | 0.29 (0.02, 0.69) |
|  | *p* value | | 0.178 | 0.233 |
| Notes: ^W^ denotes that an estimate was based on Wald’s statistics if a bound of a confidence interval was not defined | | | | |
