# Supplementary material for: Risk of stomach cancer incidence in a cohort of Mayak PA workers occupationally exposed to ionizing radiation
Source: PLoS One. 2020 Apr 15;15(4):e0231531. doi: 10.1371/journal.pone.0231531 (PMC7159243; doi:10.1371/journal.pone.0231531)
Supplement: S4 Table — W denotes that an estimate was based on Wald’s statistics if a bound of a confidence interval was not defined. (DOCX) [file pone.0231531.s004.docx]

| Table S4 The association of stomach cancer incidence risk in the study cohort with dose from external gamma rays (linear and non-liner models, males, **SmSta-adj model**) | | | | | | | |
| --- | --- | --- | --- | --- | --- | --- | --- |
| Model | Model parameters | | | Deviation | Number of parameters | Records used | Criteria for comparison |
|  | *β_1_* | *β_2_* | *β_3_* |  |  |  |  |
| **Unadjusted for internal radiation exposure** | | | | | | | |
| Linear | 0.21 (0.01, 0.50) | − | − | 4338.609 | 10 | 532183 | − |
| Quadratic | − | 0.07 (0.01, 0.17) | − | 4338.494 | 10 | 532183 | *ΔBIC* = 0.115 |
| Linear-quadratic | 0.09 (-0.33, 0.65) | 0.04 (-0.14^W^, 0.21) | − | 4338.332 | 11 | 532183 | *p* value > 0.50 |
| Linear-exponential | 0.01 (-1.11, 1.61) | − | 0.94 (-1.11, 3.10) | 4336.969 | 11 | 532183 | *p* value = 0.200 |
| **Adjusted for internal radiation exposure** | | | | | | | |
| Linear | 0.19 (-0.02, 0.49) | − | − | 4331.658 | 15 | 532183 | − |
| Quadratic | − | 0.07 (-0.00, 0.17) | − | 4331.320 | 15 | 532183 | *ΔBIC* = 0.338 |
| Linear-quadratic | 0.05 (-0.39, 0.64) | 0.05 (-0.14^W^, 0.22) | − | 4331.274 | 16 | 532183 | *p* value > 0.50 |
| Linear-exponential | 0.01 (-0.03^W^, 2.20) | − | 1.05 (-2.33, 3.29) | 4329.522 | 16 | 532183 | *p* value = 0.144 |
| Note: ^W^ denotes that an estimate was based on Wald’s statistics if a bound of a confidence interval was not defined | | | | | | | |
