# Supplementary material for: Risk of stomach cancer incidence in a cohort of Mayak PA workers occupationally exposed to ionizing radiation
Source: PLoS One. 2020 Apr 15;15(4):e0231531. doi: 10.1371/journal.pone.0231531 (PMC7159243; doi:10.1371/journal.pone.0231531)
Supplement: S5 Table — ERRed/Gy denotes an excess relative risk per 1 Gy of stomach absorbed dose from external gamma rays; ERRid/cGy denotes an excess relative risk per 1 cGy of absorbed dose from internal alpha particles for monitored workers; ERRsur denotes an excess relative risk in categories of surrogate dose for non-monitored workers;W denotes that an estimate was based on Wald’s statistics if an bound of a confidence interval was not defined; * denotes that the category included only male workers. (DOCX) [file pone.0231531.s005.docx]

| Table S5 Excess relative risks of stomach cancer incidence in the study cohort associated with external gamma and internal alpha doses (for different lag periods and both sexes, SmInd-adj model) | | | | | | | | | | | |
| --- | --- | --- | --- | --- | --- | --- | --- | --- | --- | --- | --- |
| Estimate | | | | ERR/Gy (95%CI) for various lag periods | | | | | | | |
|  |  |  |  | 0 y | | 5 y | | 10 y | 15 y | 20 y | |
| *Model: ERR= ERRed + ERRid + ERRsur* | | | | | | | | | | | |
| ERRed /Gy | | | | 0.16 (-0.02, 0.41) | | 0.17 (-0.02, 0.43) | | 0.12 (-0.05, 0.36) | 0.12 (-0.05, 0.58^W^) | 0.16 (-0.06^W^, 0.39^W^) | |
| ERRid /cGy (males)* | | | | 0.28 (-0.68^W^, 1.58) | | 0.18 (-0.90^W^, 1.65) | | 0.07 (-1.11^W^, 1.72) | -0.17 (-1.24^W^, 1.81) | -0.26 (-1.52^W^, 1.00^W^) | |
| ERRsur | 2 | | | 0.19 (-0.20, 0.72) | | 0.19 (-0.20, 0.71) | | 0.16 (-0.22, 0.67) | 0.15 (-0.28^W^, 0.65) | 0.14 (-0.27^W^, 0.56^W^) | |
|  | 3 | | | 0.54 (-0.11, 1.45) | | 0.54 (-0.10, 1.46) | | 0.53 (-0.09, 1.42) | 0.53 (-0.21^W^, 1.40) | 0.54 (-0.18^W^, 1.25^W^) | |
|  | 4 (males)* | | | 0.41 (-0.42, 1.83) | | 0.41 (-0.42, 1.82) | | 0.37 (-0.43, 1.73) | 0.35 (-0.43, 1.69) | 0.37 (-0.63^W^, 1.38^W^) | |
|  | 5–6 (males)* | | | 1.63 (0.09, 4.16) | | 1.61 (0.08, 4.13) | | 1.60 (0.13, 4.03) | 1.58 (0.13, 3.98) | 1.63 (-0.19^W^, 3.45^W^) | |
| *Model: ERR = ERRed* | | | | | | | | | | | |
| ERRed /Gy | | | | 0.17 (-0.00, 0.40) | | 0.17 (-0.00, 0.41) | | 0.11 (-0.05, 0.33) | 0.10 (-0.06, 0.31) | 0.07 (-0.08, 0.28) | |
| *Model: ERR = ERRid + ERRsur* | | | | | | | | | | | |
| ERRid /сGy | | | 0.17 (-0.43^W^, 1.10) | | 0.10 (-0.28^W^, 1.15) | | -0.02 (-0.29^W^, 1.16) | | -0.12 (-0.76^W^, 1.23) | | -0.15 (-0.97^W^, 0.66^W^) |
| ERRsur | 2 | | 0.13 (-0.23, 0.61) | | 0.13 (-0.23, 0.60) | | 0.12 (-0.23, 0.59) | | 0.12 (-0.29^W^, 0.58) | | 0.13 (-0.26^W^, 0.52^W^) |
|  | 3 | | 0.50 (-0.10, 1.33) | | 0.49 (-0.10, 1.32) | | 0.48 (-0.11, 1.30) | | 0.48 (-0.22^W^, 1.17^W^) | | 0.52 (-0.16^W^, 1.19^W^) |
|  | 4 (males)* | | 0.31 (-0.44, 1.58) | | 0.30 (-0.45, 1.57) | | 0.30 (-0.44, 1.55) | | 0.29 (-0.45, 1.27^W^) | | 0.37 (-0.58^W^, 1.32^W^) |
|  | 5–6 (males)* | | 1.54 (0.15, 3.82) | | 1.53 (0.14, 3.80) | | 1.52 (0.14, 3.77) | | 1.52 (0.14, 3.29^W^) | | 1.64 (-0.09^W^, 3.38^W^) |
| *Model: ERR = ERRid* | | | | | | | | | | | |
| ERRid /сGy | | 0.17 (-0.43^W^, 1.10) | | | 0.10 (-0.55^W^, 1.15) | | -0.03 (-0.69^W^, 1.15) | | -0.13 (-0.58^W^, 1.22) | | -0.15 (-0.97^W^, 0.67^W^) |
| Notes: ERRed/Gy denotes an excess relative risk per 1 Gy of stomach absorbed dose from external gamma rays;  ERRid/cGy denotes an excess relative risk per 1 cGy of absorbed dose from internal alpha particles for monitored workers;  ERRsur denotes an excess relative risk in categories of surrogate dose for non-monitored workers;  ^W^ denotes that an estimate was based on Wald’s statistics if an bound of a confidence interval was not defined;  * denotes that the category included only male workers. | | | | | | | | | | | |
