# Supplementary material for: Risk of stomach cancer incidence in a cohort of Mayak PA workers occupationally exposed to ionizing radiation
Source: PLoS One. 2020 Apr 15;15(4):e0231531. doi: 10.1371/journal.pone.0231531 (PMC7159243; doi:10.1371/journal.pone.0231531)
Supplement: S1 Fig — A − Adjusted for internal radiation dose, B − Unadjusted for internal radiation dose. (DOCX) [file pone.0231531.s007.docx]

Figure S1 The association of stomach cancer incidence in the study cohort with dose from external gamma rays (linear and non-linear models SmSta-adj, only males considered). A − Adjusted for internal radiation dose, B − Unadjusted for internal radiation dose
